# Supplementary material for: Role of the YAP-1 Transcriptional Target cIAP2 in the Differential Susceptibility to Chemotherapy of Non-Small-Cell Lung Cancer (NSCLC) Patients with Tumor RASSF1A Gene Methylation from the Phase 3 IFCT-0002 Trial
Source: Cancers (Basel). 2019 Nov 21;11(12):1835. doi: 10.3390/cancers11121835 (PMC6966477; doi:10.3390/cancers11121835)
Supplement: Supplementary file 1 [file cancers-11-01835-s001.pdf]

Supplmentary Materials: A Role of the YAP-1 Transcriptional Target cIAP2 in the Differential Susceptibility of Non-Small Cell Lung Cancer (NSCLC) Patients with Tumor RASSF1A Gene Methylation to Chemotherapy from the IFCT-0002 Phase 3 Trial

Fatéméh Dubois, Maureen Keller, Julien Hoflack, Elodie Maille, Martine Antoine, Virginie Westeel, Emmanuel Bergot, Elisabeth Quoix, Armelle Lavolé, Laurence Bigay-Game, Jean-Louis Pujol, Alexandra Langlais, Franck Morin, Gérard Zalcman and Guénaëlle Levallet

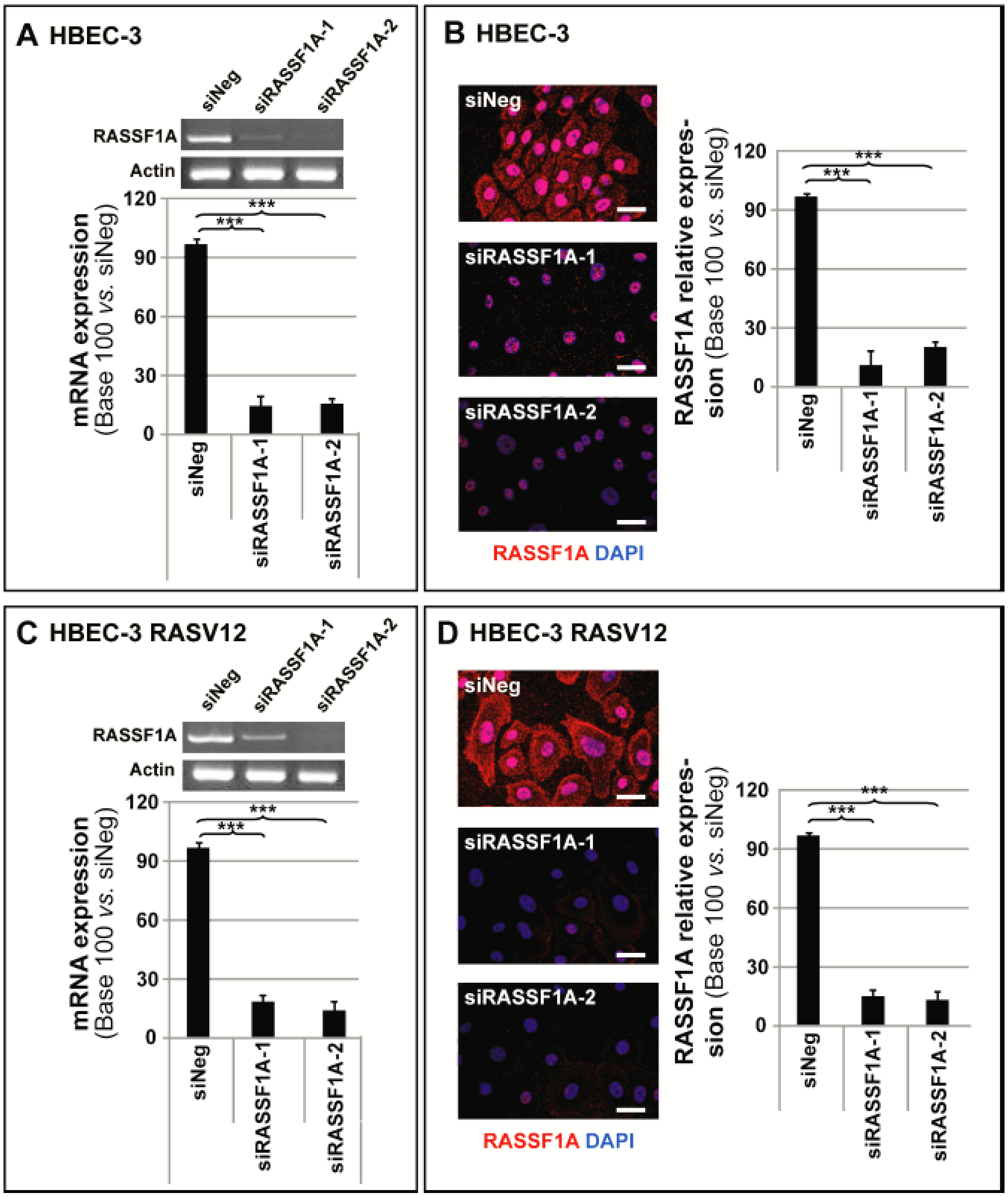

Figure S1. Evaluation of the efficiency of RASSF1A RNAi. HBEC-3 and HBEC-3 Rasv12 cells were transiently transfected with si Neg, si RASSF1A 1 & 2, Expression of RASSF1A was examined by (**A, C** RT-PCR and **B, D**) immunofluorescence experiments. The experiences were performed 48 h after transfection. For RT-PCR, actin was used as an internal control. Data are represented as the mean ± SEM from three individual experiments. Statistical significance was determined by Student's *t* test test. *** $p < 0.001$. Scale bar: 50 µm

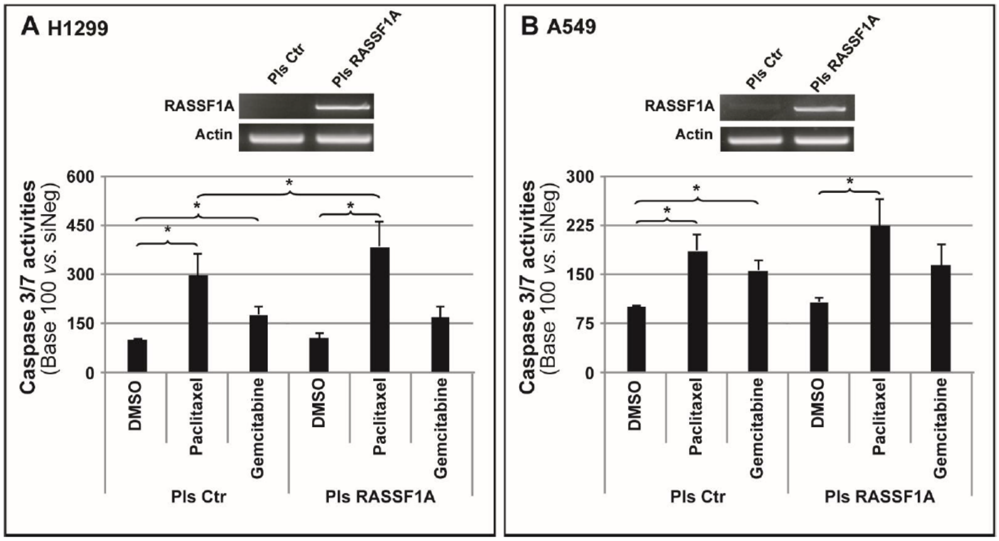

Figure S2. RASSF1A re-expression enhances cell sensitivity to drug-induced apoptosis. H1299 and A549 cells were transfected with plasmid coding wild type RASSF1A. 24 h post-transfection cells were treated, when indicated, for a further 24 h with paclitaxel (10 nM) or gemcitabine (250 nM). The experiences were performed 48 h after transfection. (**A-B**) Effect of RASSF1A re-expression on caspase-3/7 activity was measured by Caspase-Glo® 3/7 Assay kit in (**A**) H1299 and (**B**) A549 cells undergoing apoptosis by either paclitaxel or gemcitabine treatment. Efficiency of RASSF1A encoding plasmid was examined by RT-PCR as presented in upper sides of the graphs. For RT-PCR, actin was used as an internal control. Data are represented as the mean ± SEM from three individual experiments. Statistical significance was determined by Student's *t* test test. * $p < 0.05$.

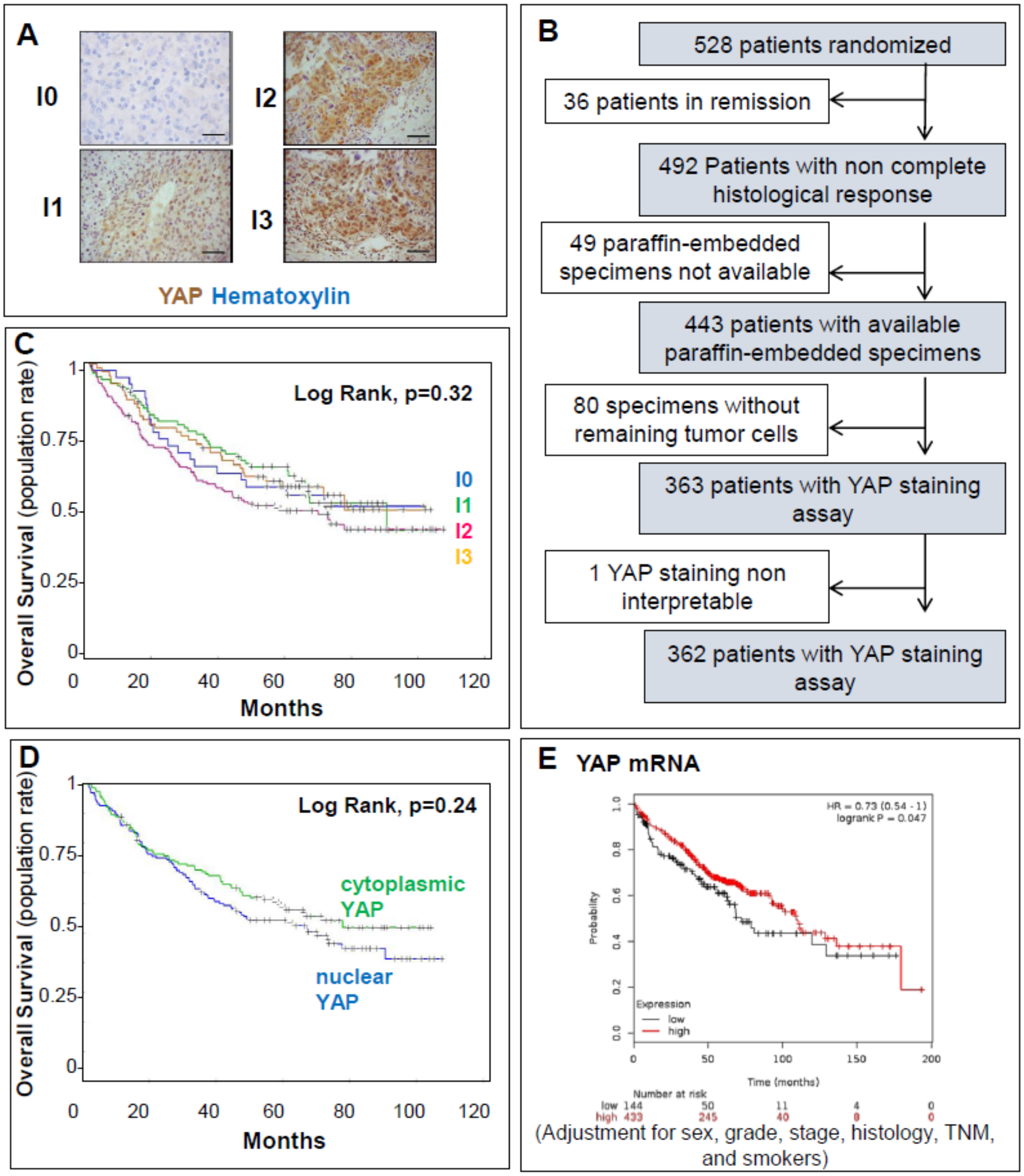

Figure S3. Relationship between Yap cytoplasmic or nuclear expression and the rates of overall survival. (**A**) Representative intensity of YAP expression measured by IHC (example of score assignment: negative (I = 0), weak (I = 1), moderate (I = 2) and strong (I = 3)). Scale bars: 200 µm. (**B**) Flowchart of patient selection and inclusion (**C-D**) Survival analyses in relation to nuclear or cytosolic YAP expression in NSCLC. (**E**) Survival analysis in NSCLC patients in relation to YAP expression using the Cancer Genome Atlas cohort.

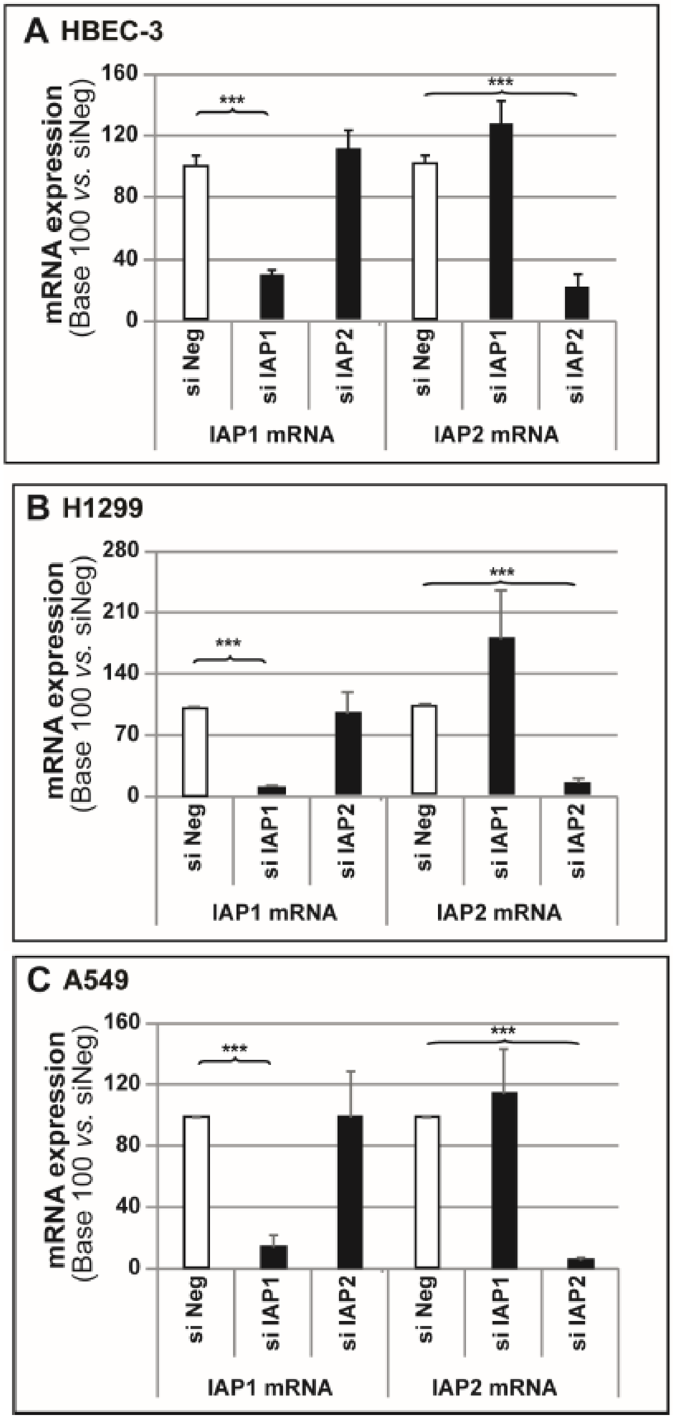

Figure S4. Evaluation of efficiency of IAP-1 and IAP-2 RNAi. The cells were transiently transfected with siRNA targeting IAP-1, IAP-2 and corresponding non-targeting control siRNA (siNeg). (**A, B, C**) Quantification of mRNA by RT-PCR indicates the efficiency of IAP-1 and IAP-2 depletion in (**A**) HBEC-3 (**B**) H1299 and (**C**) A549. S16 was used as an internal control. Data are represented as the mean ± SEM from three individual experiments. Statistical significance was determined by Student's *t* test test. *** $p < 0.001$.

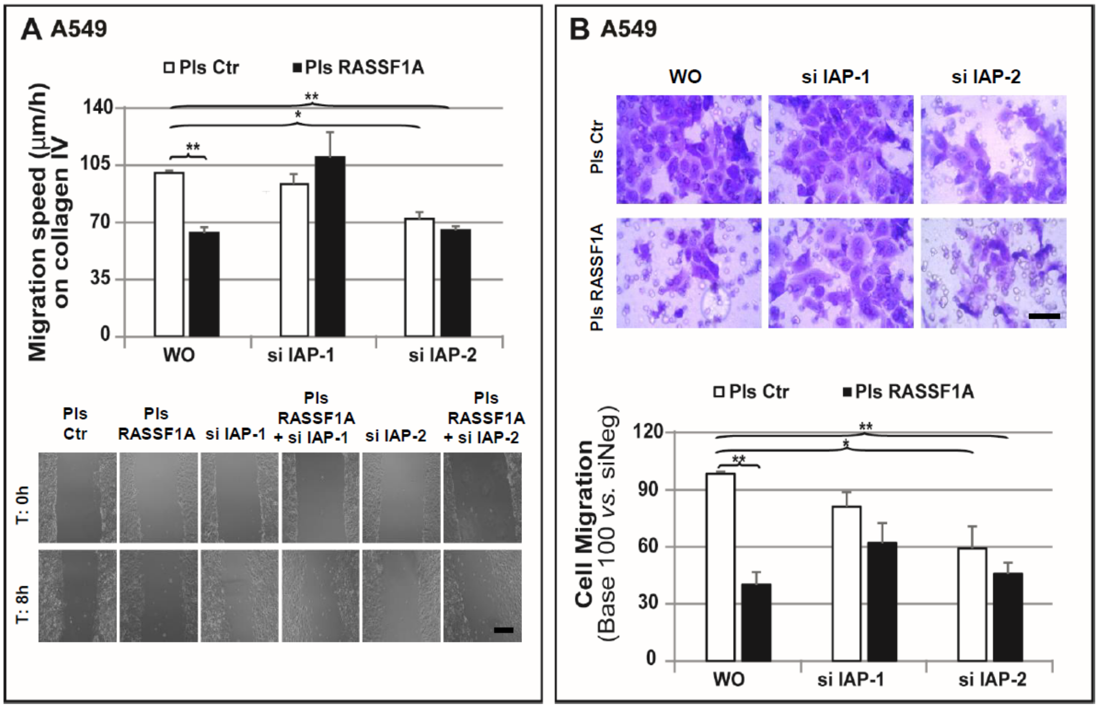

Figure S5. Increase of IAP-2 expression is critical for RASSF1A-mediated effects on cell migration and invasion. A549 cells were transfected with plasmid coding wild type RASSF1A in combination or not with siRNA targeting IAP-1, IAP-2, (as indicated on x-axis). (**A**) Migration speed (µm/h) was assessed by the wound repair assay. Scale bar, 200 µm. (**B**) 3D Migration capacity was measured by using transwell without any coating. Relative invasion normalized to that of the cells transfected with control mimic plasmid. Scale bar, 50 µm. Data are represented as the mean ± SEM from three individual experiments. Statistical significance was determined by Student's *t* test test. * $p < 0.05$; ** $p < 0.01$.

© 2019 by the authors. Submitted for possible open access publication under the terms and conditions of the Creative Commons Attribution (CC BY) license (http://creativecommons.org/licenses/by/4.0/).
